# Foreign Bodies in the Esophagus, with Report of Two Cases

**Published:** 1913-09

**Authors:** Cornelius G. Coakley

**Affiliations:** New York


### Foreign Bodies in the Esophagus, With Report of Two Cases.

The first patient, a boy 16 years of age, thought he had swallowed a piece of plate in his soup. He had a temperature of 105 degrees, rapid pulse and pneumonic area in the right lung, when first seen. Examination of the pharynx showed edema extending from the vault of the nasopharynx as far down as one could see with the laryngeal mirror, or feel with the fingers. It much resembled a retropharyngeal abscess. The X-ray showed a triangular shaped foreign body. It was jagged and evidently had cut into the mucous membrane so as rapidly to infect the pharyngeal mucosa, and the secretion from the infected pharynx passing into the larynx, setting up a septic pneumonia within twenty-four hours. The boy died fourteen hours after the operation, from acute septic pneumonia. This case shows the necessity for the prompt operative relief for removal of sharp foreign bodies. The same day I saw a boy three years of age who had swallowed a coin. The X-ray showed the coin at the level of the sixth cervical vertebra. The child had no symptoms. The coin was removed by operation and the child suffered no subsequent discomfort. In this instance the coin had been in position for five days before it was removed.—Or. Cornelius G. Coakley, New York.

